# TOOLS FOR MEASURING PERSON-CENTERED CARE FOR OLDER ADULTS IN LONG-TERM SERVICES: A SCOPING REVIEW

**DOI:** 10.1093/geroni/igad104.2300

**Published:** 2023-12-21

**Authors:** Nahida Akter, Kimberly VanHaitsma

**Affiliations:** Penn State University, State College, Pennsylvania, United States; Penn State University, State College, Pennsylvania, United States

## Abstract

Person-Centered Care (PCC) involves individuals in their care planning and decision-making. Measuring PCC is crucial in identifying the adequacy of PCC dimensions, which can then be used to promote quality of care and life, yet a comprehensive review of evidence on PCC measurement tools in long-term service and support settings (LTSS) is lacking. Our study aims to identify and evaluate available tools for measuring PCC for older adults in LTSS. This review followed the Arksey and O’Malley (2005) five-stage approach for scoping reviews and search was conducted in PubMed and CINAHL using selected keywords. The inclusion criteria were peer-reviewed journal articles written in English, reporting on PCC measurement tools for older adults (≥ 65 years) living in LTSS. Tools intended for use by family members and gray literature were excluded. Articles (n=23) were chosen from 914 studies after reviewing titles, abstracts, and full texts by two reviewers. They revealed 10 distinct tools for measuring PCC in LTSS, categorized as non-observational (e.g., interview, self-report) and observational (e.g., structured observation). The psychometric properties (reliability and validity) of the tools were reported, and their applicability in different cultural contexts was discussed. Many tools (n=8) demonstrated good reliability and validity, and their applicability was evident in multiple countries (e.g., Spain, Japan, Sweden, China, Italy and Norway). Our study adds to the discussion on PCC measurement tool development as it identifies gaps (e.g., lack of harmony between tools) for future researchers and potential areas for development by policy makers and administrators.

